# Supplementary material for: Isolation and Characterization of Bacillus Strains from Egyptian Mangroves: Exploring Their Endophytic Potential in Maize for Biological Control of Spodoptera frugiperda
Source: Biology (Basel). 2024 Dec 17;13(12):1057. doi: 10.3390/biology13121057 (PMC11673426; doi:10.3390/biology13121057)

Table S1: Major compounds in the secondary metabolites of *Bacillus tequilensis* isolate

RT	Compound Name	Area %	MF	Molecular Formula
16.64	1,4-Benzenediol, 2-(1,1-dimethylethyl)-5-(2-pr openyl)-	3.32	962	C13H18O2
20.83	Dodecyl acrylate	1.88	851	C15H28O2
23.21	1-Hexadecanol	2.71	878	C16H34O
23.41	1-Dodecanol, 3,7,11-trimethyl-	1.15	768	C15H32O
24.76	3-Isobutylhexahydropyrro lo[1,2 a]pyrazine-1,4-dione #	2.21	850	C11H18N2O2
24.93	3-Isobutylhexahydropyrro lo[1,2-a]pyrazine-1,4-dione #	1.78	846	C11H18N2O2
25.60	Cyclopropanebutanoic acid, 2-[[2-[[2-[(2 pentylcyclopropyl)methyl]cyclopropyl]methyl]cyclo propyl] methyl]-, methyl ester	1.71	724	C25H42O2
26.42	Hexadecanoic acid	5.12	789	C16H32O2
27.14	1-Nonadecene	8.09	952	C19H38
27.32	Eicosane	2.52	901	C20H42
28.60	9,12-Octadecadienoic acid (z,z)-, methyl ester	1.83	803	C19H34O2
28.78	9-Octadecenoic acid (z)-, methyl ester	2.88	853	C19H36O2
29.34	Methyl-9,9,10,10-d4-octadeca noate	1.39	743	C19H34D4O2
30.75	1-Docosene	9.69	933	C22H44
30.90	Docosane	2.80	833	C22H46
34.07	Nonacos-1-ene	6.77	933	C29H58
34.20	Dotriacontane	2.31	797	C32H66
34.58	Cholestan-3-ol, 2-methylene-, (3á,5à)-	4.55	792	C28H48O
34.72	8-Azabicyclo[3.2.1]octan-3- ol, 8-methyl-, benzoate (ester), exo-	1.08	697	C15H19NO2
34.88	Isochiapin b	1.00	707	C19H22O6
35.18	1-Heptatriacotanol	1.00	719	C37H76O
35.77	Diisooctyl phthalate	9.05	952	C24H38O4
36.20	11-Octadecenal (spectrum disagrees)	1.36	687	C18H34O
36.30	11-Octadecenal (spectrum disagrees)	1.26	702	C18H34O
36.47	2,2-Dideutero octadecanal	2.23	690	C18H34D2O
36.76	11-Octadecenal (spectrum disagrees)	1.40	700	C18H34O
37.14	2-Hexadecanol	3.04	802	C16H34O
37.26	Dotriacontane	1.11	775	C32H66
38.01	9,10 Dideutero octadecanal	1.52	696	C18H34D2O
38.74	Dotriacontane	1.47	754	C32H66
39.99	Hahnfett	1.74	698	N/A
40.06	Dotriacontane	1.35	723	C32H66
40.99	Dotriacontane	3.87	725	C32H66
41.87	9,12-Octadecadienoic acid (z,z)-, 2,3-bis[(trimethylsilyl)oxy]propyl ester	1.70	730	C27H54O4Si2
41.94	9,12-Octadecadienoic acid (z,z)-, 2,3-bis[(trimethylsilyl)oxy]propyl ester	0.81	729	C27H54O4Si2
43.03	Arabinitol, pentaacetate	2.31	693	C15H22O10

Table S2: Major compounds in the secondary metabolites of ***Bacillus* spp** isolate

RT	Compound Name	Area %	MF	Molecular Formula
16.65	3,4-Dihydro-2h-1,5-(3"-t-butyl)benzodioxepine	0.82	920	C13H18O2
18.85	5-Fluoro-2,2-dimethylchro man-4-one	2.58	834	C11H11FO2
20.84	9-Hexadecenoic acid	1.82	771	C16H30O2
24.76	1,3-Propanediol, 2-methyl-2-(1-methylpropy l)-, dicarbamate	1.09	638	C10H20N2O4
26.49	Hexadecanoic acid, 2,3-dihydroxypropyl ester	3.73	784	C19H38O4
27.15	Nonacos-1-ene	3.83	933	C29H58
27.32	2-Hexadecanol	1.74	785	C16H34O
28.79	6-Octadecenoic acid	1.37	812	C18H34O2
29.34	Methyl-9,9,10,10-d4-octadeca noate	0.62	757	C19H34D4O2
30.75	Nonacos-1-ene	7.18	925	C29H58
30.91	Docosane	2.31	819	C22H46
34.07	2-Hexadecanol	5.20	829	C16H34O
34.21	Heptacosane	2.35	803	C27H56
34.58	Cholestan-3-ol, 2-methylene-, (3á,5à)-	4.07	785	C28H48O
34.72	1-Heptatriacotanol	0.87	716	C37H76O
34.88	Isochiapin b	0.92	706	C19H22O6
35.18	Dotriacontane	1.01	706	C32H66
35.77	Diisooctyl phthalate	8.11	947	C24H38O4
36.20	11-Octadecenal (spectrum disagrees)	1.54	679	C18H34O
36.30	Dotriacontane	1.16	710	C32H66
36.48	Isochiapin b	2.22	690	C19H22O6
36.76	1-Heptatriacotanol	1.03	716	C37H76O
37.14	2-Hexadecanol	2.62	795	C16H34O
37.27	Dotriacontane	1.03	757	C32H66
37.34	Dotriacontane	0.61	709	C32H66
37.70	1-Heptatriacotanol	0.92	702	C37H76O
38.01	1-Heptatriacotanol	0.97	703	C37H76O
39.99	Cis-13-eicosenoic acid	1.50	710	C20H38O2
40.06	Dotriacontane	1.39	735	C32H66
40.99	9,12-Octadecadienoic acid (z,z)-, 2,3 bis[(trimethylsilyl)oxy]propyl ester	1.49	735	C27H54O4Si2
41.39	9,12-Octadecadienoic acid (z,z)-, 2,3-bis[(trimethylsilyl)oxy]propyl ester	0.96	730	C27H54O4Si2
41.56	9,12-Octadecadienoic acid (z,z)-, 2,3-bis[(trimethylsilyl)oxy]propyl ester	0.87	719	C27H54O4Si2
41.87	9,12-Octadecadienoic acid (z,z)-, 2,3-bis[(trimethylsilyl)oxy]propyl ester	3.28	721	C27H54O4Si2
42.87	9,12-Octadecadienoic acid (z,z)-, 2,3-bis[(trimethylsilyl)oxy]propyl ester	20.60	716	C27H54O4Si2
43.22	Arabinitol, pentaacetate	8.16	691	C15H22O10

Table S3: Major compounds in the secondary metabolites of *Bacillus subtilis* isolate

RT	Compound Name	Area %	MF	Molecular Formula
16.63	3,4-Dihydro-2h-1,5-(3"-t-butyl)benzodioxepine	5.77	960	C13H18O2
18.78	5-Fluoro-2,2-dimethylchro man-4-one	4.77	855	C11H11FO2
20.80	Dodecyl acrylate	1.51	863	C15H28O2
23.20	1-Nonadecene	2.58	935	C19H38
23.40	Tetradecane, 2,6,10-trimethyl-	1.23	834	C17H36
24.75	7,9-Di-tert-butyl-1-oxaspiro(4,5)dec a-6,9-diene-2,8-dione	1.27	842	C17H24O3
25.41	Benzoic acid, pentachloro-	0.59	965	C7HCl5O2
25.59	Hexadecanoic acid, methyl ester	1.24	841	C17H34O2
26.39	Hexadecanoic acid	4.07	886	C16H32O2
26.95	1-Dodecanol, 3,7,11-trimethyl-	0.65	765	C15H32O
27.14	1-Docosene	6.50	951	C22H44
27.31	Hexatriacontane	2.61	864	C36H74
28.58	12-Methyl-e,e-2,13-octadecadien-1- ol	1.43	819	C19H36O
28.77	9-Octadecenoic acid (z)-, methyl ester	1.74	869	C19H36O2
29.33	Methyl-9,9,10,10-d4-octadeca noate	1.19	698	C19H34D4O2
30.06	Octahydro-1,8a(1h)-naphth alenediol	1.02	790	C10H18O2
30.75	1-Docosene	7.75	942	C22H44
30.90	Docosane	2.61	859	C22H46
33.75	Dotriacontane	0.66	735	C32H66
34.07	Nonacos-1-ene	5.33	941	C29H58
34.20	Heptacosane	2.00	844	C27H56
34.57	Cholestan-3-ol, 2-methylene-, (3á,5à)-	3.10	792	C28H48O
34.87	1,3,5-Triazine-2,4-diamine, 6-chloro-n-ethyl-	0.96	754	C5H8ClN5
35.17	1-Heptatriacotanol	0.96	693	C37H76O
35.76	Diisooctyl phthalate	9.56	955	C24H38O4
36.20	1-Heptatriacotanol	1.17	703	C37H76O
36.29	Dotriacontane	1.18	721	C32H66
36.46	Carbonic acid, octadecyl phenyl ester	2.42	860	C25H42O3
36.75	11-Octadecenal (spectrum disagrees)	1.05	701	C18H34O
37.14	1-Hexacosene	2.67	821	C26H52
37.26	Dotriacontane	1.73	808	C32H66
37.70	1-Heptatriacotanol	0.76	730	C37H76O
38.01	1-Heptatriacotanol	1.22	708	C37H76O
39.98	2-Hexadecanol	1.89	778	C16H34O
40.06	Dotriacontane	1.30	778	C32H66
40.99	4h-1-Benzopyran-4-one, 2-(3,4 dimethoxyphenyl)-3,5-dihydroxy-7-methoxy-	0.85	732	C18H16O7
41.86	Oleic acid, 3-(octadecyloxy)propyl ester	3.18	690	C39H76O3
42.85	9,12-Octadecadienoic acid (z,z)-, 2,3-bis[(trimethylsilyl)oxy]propyl ester	6.96	719	C27H54O4Si2
43.22	2-Bromotetradecanoic acid	2.51	715	C14H27BrO2

Figure. S1. Plant height (cm) of maize as affected by three plant-colonizing bacterial isolates using different inoculation methods: seed treatment, soil drench, foliar application, and combined methods at 20-, 30- and 40-days post-inoculation.

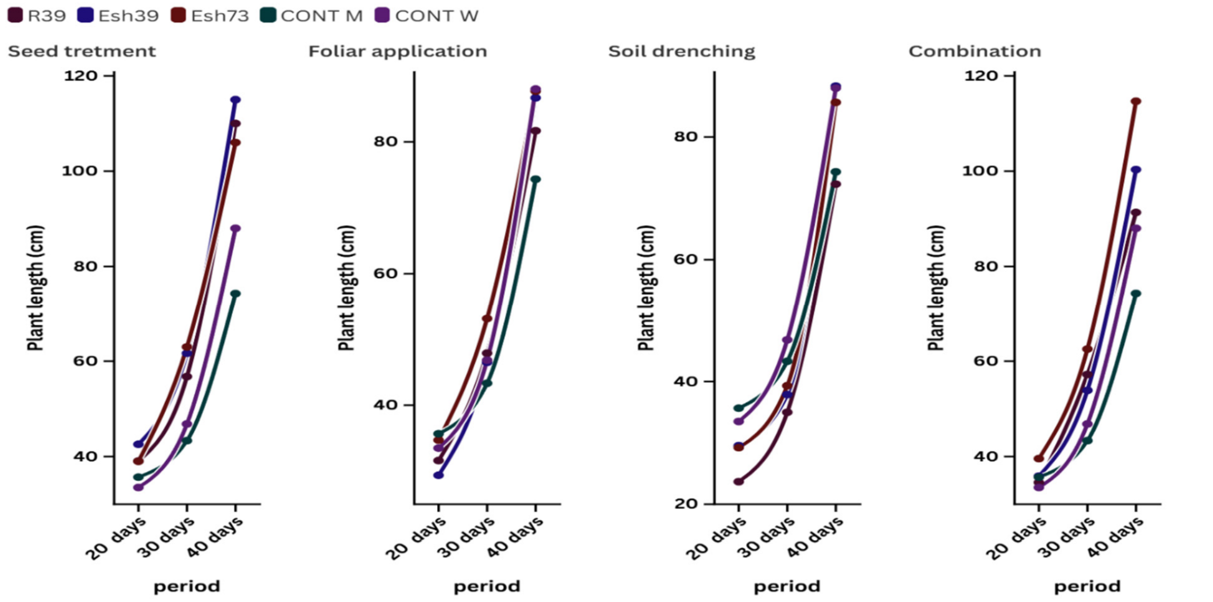

Figure S2. Chlorophyll content (mg/m-2) in maize plant as affected by three plant-colonizing bacterial isolates using different inoculation methods: seed treatment, soil drench, foliar application, and combined method at 30- and 40-days post-inoculation.

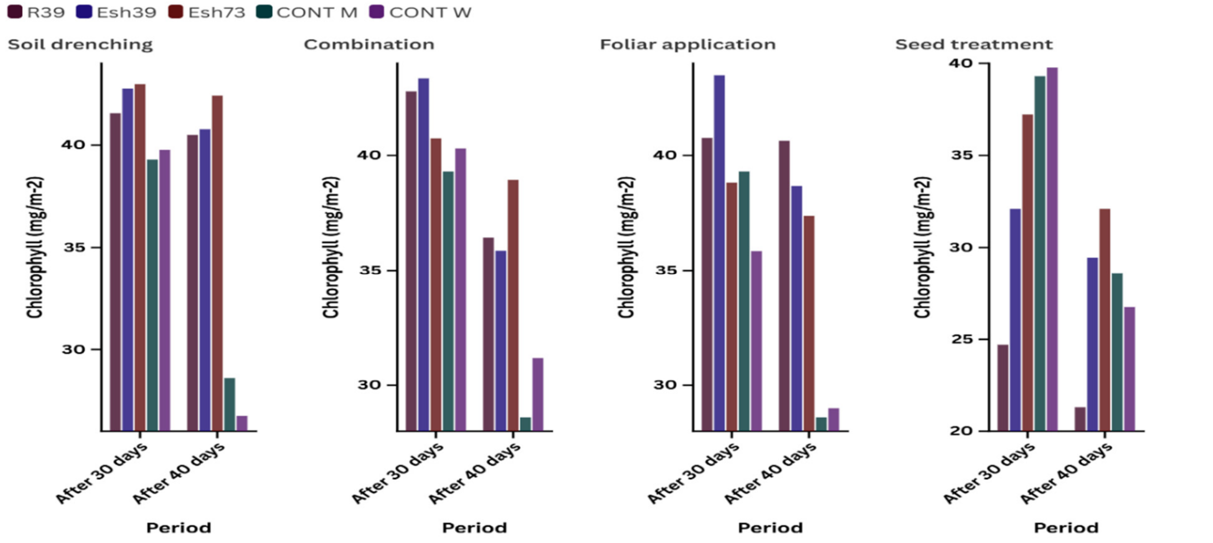

Supplement: Supplementary file 1 [file biology-13-01057-s001.zip › biology-3316024-supplementary.pdf]
